# Establishment of in-hospital nutrition support program for middle-aged and elderly patients with acute decompendated heart failure

**DOI:** 10.1186/s12872-024-03887-y

**Published:** 2024-05-18

**Authors:** Yongliang Li, Fang Zhu, Dongmei Ren, Jianping Tong, Qin Xu, Minhui Zhong, Wei Zhao, Xia Duan, Xiangdong Xu

**Affiliations:** 1 CCU, Jiading District Central Hospital Affiliated Shanghai University of Medicine & Health Sciences, Shanghai, 201800 China; 2 Department of Nursing, Jiading District Central Hospital Affiliated Shanghai University of Medicine & Health Sciences, Shanghai, 201800 China; 3 Department of Cardiovascular Medicine, Jiading District Central Hospital Affiliated Shanghai University of Medicine & Health Sciences, Shanghai, 201204 China; 4 Department of Emergency, Jiad-ing District Central Hospital Affiliated Shanghai University of Medicine &Health Sciences, Shanghai, 201800 China; 5 Department of Nursing, Shanghai Key Laboratory of Maternal Fetal Medicine, Shanghai Institute of Maternal-Fetal Medicine and Gynecologic Oncology, Shanghai First Maternity and Infant Hospital, School of Medicine, Tongji University, Shanghai, 200092 China; 6 Suzhou Science & Technology Town Hospital, Tongji University School of Medicine, Shanghai, 200120 China

**Keywords:** Acute decompensated heart failure, Nutrition support, Delphi process

## Abstract

**Objective:**

To construct a nutrition support program for middle-aged and elderly patients with acute decompensated heart failure (ADHF) during hospitalization.

**Methods:**

Based on the JBI Evidence-Based Health Care Model as the theoretical framework, the best evidence was extracted through literature analysis and a preliminary nutrition support plan for middle-aged and elderly ADHF patients during hospitalization was formed. Two rounds of expert opinion consultation were conducted using the Delphi method. The indicators were modified, supplemented and reduced according to the expert’s scoring and feedback, and the expert scoring was calculated.

**Results:**

The response rates of the experts in the two rounds of consultation were 86.7% and 100%, respectively, and the coefficient of variation (CV) for each round was between 0.00% and 29.67% (all < 0.25). In the first round of expert consultation, 4 items were modified, 3 items were deleted, and 3 items were added. In the second round of the expert consultation, one item was deleted and one item was modified. Through two rounds of expert consultation, expert consensus was reached and a nutrition support plan for ADHF patients was finally formed, including 4 first-level indicators, 7 s-level indicators, and 24 third-level indicators.

**Conclusion:**

The nutrition support program constructed in this study for middle-aged and elderly ADHF patients during hospitalization is authoritative, scientific and practical, and provides a theoretical basis for clinical development of nutrition support program for middle-aged and elderly ADHF patients during hospitalization.

## Introduction

Heart failure (HF) is a common cardiovascular disease, which is the final stage of various cardiac lesions [1]. It is characterized by abnormal cardiac structure or function that impairs ventricular filling or ejection capacity, resulting in pulmonary congestion or systemic congestion, with or without tissue organ hypoperfusion [2]. Acute decompensated heart failure (ADHF) refers to the rapid onset or worsening of symptoms and signs due to cardiac dysfunction, accompanied by elevated plasma natriuretic peptide levels [3]. ADHF is a common emergency condition that requires rapid diagnosis and urgent treatment, otherwise it may lead to serious complications and death [4]. The elderly population is a high-risk group for heart failure, and the incidence and mortality of heart failure increase with age [5]. Elderly ADHF patients often have multiple comorbidities and precipitating factors, such as hypertension, coronary heart disease, diabetes, renal insufficiency, anemia, infection, etc., which increase the complexity and difficulty of treatment [6–8]. In addition, elderly ADHF patients are prone to malnutrition or deterioration during hospitalization, affecting prognosis and quality of life [9].

Malnutrition and physical decline are common in elderly heart failure patients, and both are associated with adverse outcomes [10, 11]. Kałużna-Oleksy et al. has revealed that up to half of the patients with HF with reduced ejection fraction are at risk of malnutrition, with 2.9% suffering from it, and no significant differences in nutritional status between genders [12]. Studies have shown that malnutrition is an independent risk factor for the prognosis of heart failure patients, and ADHF patients have a malnutrition rate of 75-90% [13, 14]. Effective nutrition support is essential for the recovery of ADHF patients during hospitalization, which can reduce the recurrence of acute decompensation of chronic heart failure, alleviate heart failure symptoms, and improve the nutritional status and prognosis of patients [15]. However, there is no standardized nutrition support protocol in clinical practice. Therefore, based on literature review and clinical expert discussion, this study uses the Delphi method to construct a nutrition support protocol for elderly ADHF patients during hospitalization, providing a theoretical basis for nutrition support for elderly ADHF patients during hospitalization.

## Methods

### Establishment of the research group

The research team consisted of one doctoral supervisor, two nursing postgraduates, one deputy chief physician of the cardiovascular department, one head nurse, and one nutritionist. The main tasks of the research team included literature search and evaluation, expert inquiry form development, expert selection, expert recommendation analysis and integration, and protocol determination.

### Literature search and selection criteria

Following the Evidence Pyramid “6S” model, we searched domestic and international guideline websites, databases and related websites, including BMJ Best Practice, Up To Date, Guidelines International Network (GIN), National Guideline Clearinghouse (NGC), Medlive (Yi Mai Tong), Cochrane Library, Joanna Briggs Institute, PubMed, Scottish Intercollegiate Guidelines Network (SIGN), National Institute for Health and Clinical Excellence (NICE), National Clinical Practice Guidelines Database (NGC), China Science and Technology Journal Database, Wanfang, China National Knowledge Infrastructure (CNKI), and China Biomedical Literature Database for studies on nutritional plans for acute and chronic heart failure patients published from the inception of each database to December 2021. The search terms included “heart failure; chronic heart failure, acute heart failure, acutely decompensated chronic heart failure, nutri*, malnutrition, dietary, diet, micronutrients, vitamins, trace elements”. The language of the literature was limited to English and Chinese. To ensure the comprehensiveness of the search, we also screened the references of the retrieved literature.

The inclusion criteria for the literature were: (1) Chinese or English language. (2) Clinical practice guidelines, evidence summaries, best evidence, systematic reviews, expert consensus. (3) Providing guiding opinions on clinical nutrition support. The exclusion criteria for the literature were: (1) Literature with a quality assessment of grade C; (2) Duplicated publications; (3) Literature with unavailable full text; (4) Old guidelines that have been replaced.

### Evaluation of the quality of the literature

This study used the evidence-based method of the Australian JBI Evidence-Based Health Care Center (2014), and two people who had received evidence-based course training conducted evidence extraction, analysis and synthesis of the included literature, and prepared a draft of the nutrition support program for middle-aged and elderly ADHF patients during hospitalization. The program included four parts: team organization and management, nutritional assessment, nutritional intervention measures, and outcome evaluation. In case of disagreement, the two people reached a consensus through discussion and consultation.

### Expert selection criteria

This study invited experts from Shanghai, Suzhou, and Anhui in China. Experts were selected based on their clinical expertise and previous publications in the field of cardiovascular disease and nutrition. From July to October 2022, the members of the research team conducted expert consultation through email and WeChat. The inclusion criteria for the experts were as follows: (1) Senior specialists with 10 or more years of nursing or clinical experience in cardiovascular medicine, critical care disciplines, and geriatrics; (2) Working years of 10 years or more; (3) Bachelor degree or above, and the title of intermediate or above; (4) Agree to participate in two rounds of inquiry and provide modification suggestions for improving the plan.

### Establishing the questionnaire

The questionnaire consists of three parts. (1) Questionnaire instructions: The experts are introduced to the research background, purpose, significance and filling method. (2) Expert basic information: The general information of the experts includes gender, age, working years, professional title, education level, work unit, work field, etc. Experts’ familiarity and judgement basis self-evaluation table: Familiarity is divided into very unfamiliar (0.2), unfamiliar (0.4), generally familiar (0.6), familiar (0.8), and very familiar (1). The judgement basis is classified into four categories: practical experience, theoretical analysis, reference to domestic and foreign literature, and intuition selection. The degree of influence is divided into large, medium, and small, and different quantitative values are assigned accordingly. (3) Expert inquiry form on nutrition support plan for elderly ADHF patients during hospitalization: The importance of the strategy recommendation was rated using the Likert 5-point scoring method: “5” for “extremely important”, “4” for “very important”, “3” for “moderately important”, “2” for “slightly important” and “1” for “unimportant”. In addition, each item has a modification comment column for the experts to modify, delete or add items.

### Implementation of an expert consultation

A two-round online Delphi survey was conducted from July to October 2022. After the first round of expert consultation letters were collected, the research team members sorted, counted and analyzed the expert opinions and provided feedback to the experts. According to the item modification principles, they added or deleted relevant item contents and formed the second round of expert consultation letters. Following the same process, they completed the subsequent consultation for the “Nutrition support Plan for Elderly ADHF Patients During Hospitalization” until the expert opinions converged and the consultation was stopped. Based on the importance scores of the items, the principles for modifying the items were formulated in accordance with the clinical work needs. The items with a mean value ≥ 4 and a coefficient of variation ≤ 0.25 were retained. At the same time, to avoid deleting important items, for those items that only had one criterion not met, or that met both criteria but had large deviations in some experts’ opinions, the principles of science and practicality were followed, and the experts’ opinions were respected. The decision to keep or discard the items was made through collective discussion.

### Statistics analysis

SPSS 26.0 software is used for data statistics and analysis. The effective recovery rate of the consultation form represents the enthusiasm of the experts. The expert authority coefficient (CR) is calculated based on the average of the expert judgment foundation (Ca) and familiarity (CS) of the research plan, that is, CR=(Ca + CS)/2. The degree of coordination of expert opinions is expressed by Kendall’s harmony coefficient (Kendall´s W) and coefficient of variation CV.

## Results

### Results of the literature search

**Fig. 1:**
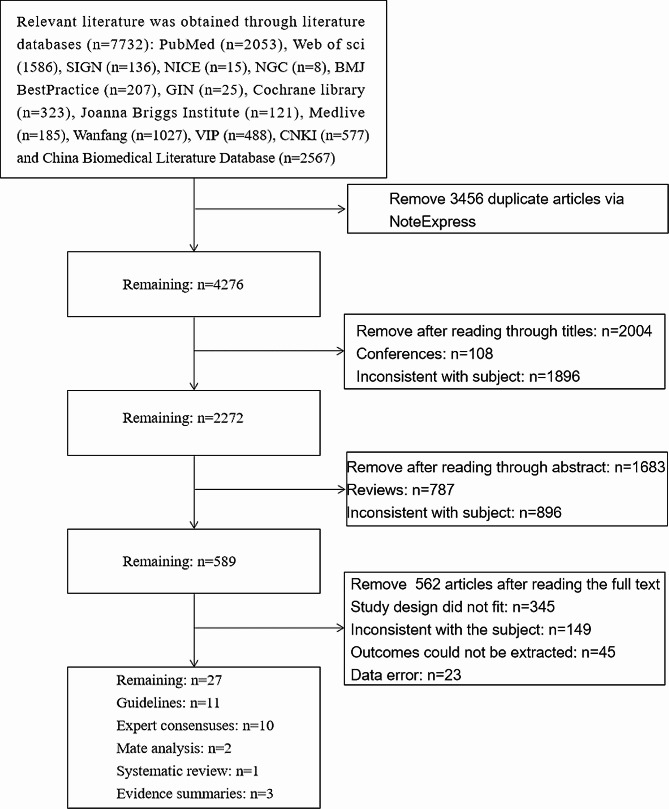
Flow chart of the process of selecting article for scoping review. SIGN: Scottish Intercollegiate Guidelines Network, NICE: National Institute for Health and Clinical Excellence, NGC: National Guideline Clearinghouse, GIN: Guidelines International Network, VIP: China Science and Technology Journal Database, CNKI: China National Knowledge Infrastructure

This study retrieved 7732 relevant articles, and after removing duplicates, screening titles and abstracts, 7701 articles were excluded, and 31 articles were preliminarily included. After further reading the full text, 2 articles without recommendation strength, 1 article with an updated version, and 1 article with weak relevance were excluded, and 27 articles [1, 13, 16–40] were finally included (Fig. 1). The included articles consisted of 11 guidelines, 10 expert consensuses, 1 systematic review, 2 Mate analysis and 3 evidence summaries.

### Basic information of experts

This study invited 15 experts from different fields, including 6 cardiovascular clinicians, 1 clinical nutritionist, 1 clinical dietitian, 6 clinical nursing and management experts in cardiology and 1 rehabilitation therapist. The experts had work experience ranging from 11 to 27 years, with bachelor’s degree or above, and intermediate or above professional titles (Table 1).

**Table 1:** Basic characteristics of experts

Items	Number	Percent (%)
Gender		
Male	8	53.33
Female	7	46.67
Major/Department		
Nursing	6	40
Nutrition	2	13.33
Doctor	6	40
Rehabilitation	1	0.67
Working years	15	67.54
Professional title		
Senior	2	13.33
Deputy senior	6	40
Intermediate	7	46.67
Degree of education		
Doctoral Degree	3	20
Master’s degree	4	26.67
Bachelor’s degree	8	53.33

### Enthusiasm and authority of the experts

The Cr of the experts reflects the reliability of the consultation results. Cr is calculated based on the average of the experts’ judgment foundation (CA) and familiarity (CS) of the research plan. It is generally believed that when Cr ≥ 0.7, the consultation results have a high credibility, and the higher the score, the higher the credibility. The two rounds of Cr in this study were 0.87 and 0.89, respectively, indicating that the expert opinions in this study had a high degree of authority.

### Degree of coordination of the expert opinions

After two rounds of consultation, the coefficient of variation CV for each round was between 0.00% and 29.67% (all < 0.25). In addition, the Kendall’s coefficient of concordance Kendall’s W for the first and second round questionnaires were 0.323 and 0.398, respectively, *P* < 0.05, indicating that the experts had a high degree of consensus on the evaluation results of the indicators, and the evaluation results were reliable.

### Selection of indicators

In the first round of expert consultation, 4 items were modified, 3 items were deleted, and 3 items were added. (1) Modifications: The term “nutritional assessment” was revised to “nutritional screening and assessment”. The rationale for this change is that screening and assessment are distinct actions; patients are first subjected to nutritional screening upon admission, and if at risk of malnutrition, they proceed to a comprehensive nutritional assessment. The term “formation of a heart failure nutrition support team: cardiologists, geriatricians, heart failure nurse practitioners, dietitians, cardiac rehabilitators, and counselors form the team” was changed to “mainly by heart failure specialists and dietitians, in collaboration with critical care physicians, geriatricians, heart failure specialist nurses, and cardiac rehabilitation therapists to form a heart failure nutrition support team”. This modification was made to clarify the core members of the multidisciplinary team, facilitating decisive adjustments to the dynamic treatment plan. “Fluid management” was updated to “volume management”, as the former term tends to focus on the intake, infusion, and output of fluids, whereas “volume management” encompasses a broader concept including systemic and pulmonary circulatory volume loads. The “weight loss diet” was revised to “low-calorie diet”, with the reasoning that the former is subjectively aimed at reducing fat or body weight, while the latter is an objective dietary pattern controlling caloric intake due to excess energy and nutrition, aligning more closely with medical interventions. (2) Deletions: The recommendation to “reduce the intake of caffeinated beverages and alcohol for ADHF patients” was removed, as the consumption of coffee and alcohol is not applicable to clinical inpatients. “Cachexia enteral nutrition” was deleted because heart failure patients with cachexia were already excluded from the inclusion criteria, and the significance of nutritional support for patients who have progressed to cachexia remains debatable. The team “eating 30 g of unsalted nuts daily” was also removed, as the high fatty acid content in nuts contradicts the low-fat diet recommended for heart failure patients. (3) Additions: The inclusion of “non-pharmacological measures to alleviate symptoms of dry mouth and thirst” is proposed. The rationale is that patients with cardiac insufficiency commonly experience xerostomia and intense thirst, which contradicts the restrictions on fluid intake. It is recommended to employ non-pharmacological strategies to improve patient comfort and alleviate these symptoms. The creation of “standardized educational materials” is advised. This is due to the observed variability in the understanding and communication of nutritional education among different healthcare professionals. Standardized and unified health education is anticipated to significantly enhance the guidance provided to patients and their families. Furthermore, the outcome indicators focus on nutritional biochemical markers. Experts recommend the inclusion of “prealbumin (PAB)” and ”albumin (ALB)” in the “blood test report” to enhance the sensitivity of these indicators. After discussion by the project team, the experts’ suggestions were adopted.

In the second round of expert consultation, it was recommended to remove the term “plant-based diet”-a regimen predominantly composed of plant-derived foods, encompassing fruits, vegetables, whole grains, and nuts, and is often synonymous with a vegan diet [41, 42]. The reasons for this decision stems from clinical observations that plant-based diets frequently induce gastrointestinal bloating. This is particularly pertinent for patients with heart failure, who commonly experience gastrointestinal congestion, as such bloating can impede nutritional absorption. Furthermore, experts proposed an amendment to the “vassessment time within 24 hours”, suggesting it be “completed within the shift or 6 hours” to ensure prompt intervention for patients at nutritional risk. After discussion by the project team, these suggestions were adopted. Furthermore, an expert recommended the removal of “the assessment and supplementation of iron” from the protocol, citing its infrequent application in clinical practice. The research team, after reviewing and discussing relevant literature, concluded that iron deficiency can adversely affect the exercise capacity, quality of life, and functional status of patients with ADHF. Moreover, a report indicated that the prevalence of iron deficiency was 68.6% in male patients with heart failure, and even higher in females, at 75.3% [43]. After careful consideration of the AHA/ACC/HFSA 2022 guidelines [44] and the significance attributed to this indicator, the team has resolved not to incorporate this recommendation. After two rounds of expert consultation, the experts reached a consensus, and finally formed a nutrition support program for ADHF patients, including 4 primary indicators, 7 secondary indicators, and 24 tertiary indicators. The mean and coefficient of variation CV of the importance of each item are shown in Table 2.

**Table 2:** Nutrition support program and importance rating of items for elderly ADHF patients (x ± s) coefficient of variation

Recommendation (Implementation plan)	Score	CV (%)
I. Team organization and management	5.00 ± 0.00	0
1. A heart failure nutritional support team should be formed mainly composed of heart failure specialists and nutritionists, in conjunction with critical care physicians, geriatric physicians, heart failure specialist nurses, and cardiac rehabilitation therapists	4.46 ± 0.77	12.56
2. Employ specialist nutrition nurses to work with nutritionists, provide nutrition support training and quality control for responsible nurses	4.85 ± 0.38	13.41
3. Establish nutritional support standard procedures and clarify responsibilities	4.54 ± 0.37	13.23
4. The hospital should set up a nutrition guidance committee including medical, nursing, nutrition, pharmacology, psychology, and cardiac rehabilitation therapists	4.77 ± 0.66	11.56
II. Nutritional screening and assessment	4.96 ± 0.25	6.42
5. Nutritional risk screening and assessment should be completed for new patients on duty or within 6 h of admission	4.62 ± 0.78	14.09
6. Nutritional screening should use NRS2002, and nutritional assessment should use the Mini Nutritional Assessment for Heart Failure (MNA-HF)	4.54 ± 0.38	13.51
7. The assessment should include relevant medical history, dietary survey, physical examination (leg circumference, arm circumference, BMI, etc.), blood tests: PAB, ALB, liver and kidney function, blood sugar, blood lipids, electrolytes, acid-base balance, etc.	4.69 ± 0.38	10.97
8. Weekly evaluations and reassessments should be conducted during recurrent acute heart failure episodes	4.54 ± 0.51	11.46
III. Nutritional intervention measures	5.00 ± 0.00	0
(I) Volume management	5.00 ± 0.00	0
9. Every shift should record fluid input and output and guide patients to participate in fluid management: record water intake and defecation	4.85 ± 0.75	12.56
10. Control daily water intake + fluid replacement to about 1500-2000 ml, infusion rate < 100 ml/h	4.62 ± 0.54	14.63
11. For patients with pulmonary circulation and circulatory congestion, use diuretics, maintain a daily negative balance of about 500 ml, and monitor electrolyte balance	4.62 ± 0.53	7.76
12. Use non-pharmacological methods to improve dry mouth and thirst, such as increasing water intake and rinsing mouth with ice water spray	4.87 ± 0.63	8.93
(II) Energy management	5.00 ± 0.00	0
13. Indirect calorimetry combined with energy calculation formula to calculate total energy demand, and allocate the percentage of dietary composition (carbohydrates, proteins, fats)	4.87 ± 0.61	9.19
14. Oral nutritional supplements (ONS) should be added on the basis of diet: choose high energy density (1.5-2.0 kcal/ml)	4.79 ± 0.58	13.57
15. Standard nutritional intervention therapy: increase meal frequency, provide 400–600 kcal or 30 g protein per day	4.68 ± 0.37	14.39
16. Peripheral parenteral nutrition should be used in combination with gastrointestinal dysfunction	4.63 ± 0.59	13.87
(III) Individualized diet pattern	4.93 ± 0.42	6.57
17. Customized low-salt, low-fat, Mediterranean, low-calorie, and DASH diets for terminating hypertension	4.77 ± 0.36	13.54
18. For patients with chewing and swallowing difficulties, provide rich soft or liquid diets to ensure adequate food intake	4.91 ± 0.42	13.21
(IV) Nutrient support	5 ± 0.00	0
19. Regularly measure relevant nutrients and supplement nutrient deficiencies according to medical advice: high-quality protein, amino acids, unsaturated fatty acids, minerals (potassium, sodium, iron), coenzyme Q10, vitamins, dietary fiber, probiotics, etc.	4.68 ± 0.35	14.67
(V) Health education	5.00 ± 0.00	0
20. Standardized health education should be provided to patients and their families throughout the hospitalization period, and education manuals should be provided [16, 30]	4.72 ± 0.48	14.73
21. The responsible nurse should provide personalized nutrition counseling to patients and their families, encourage the combination of diet and exercise, and improve compliance	4.69 ± 0.67	10.11
IV. Effect evaluation	5.00 ± 0.00	0
(I) Nutritional indicators	4.87 ± 0.37	11.56
22. Physical examination (leg circumference, arm circumference, triceps skinfold thickness, BMI), assessment of abdominal distension, diarrhea, and lower limb edema	4.92 ± 0.69	10.65
23. Blood test report: PAB, ALB, liver and kidney function, blood sugar, blood lipids, electrolytes, acid-base balance, etc.	4.85 ± 0.47	12.64
(II) Cardiac function indicators	4.96 ± 0.55	10.36
24. NYHA cardiac function classification, NT-proBNP, EF value, 6MWT, etc.	4.86 ± 0.65	9.16

*Note* BMI, body mass index; PAB, prealbumin; ALB, serum albumin; NT-proBNP, N-terminal pro-brain natriuretic peptide; EF, ejection fraction; 6MWT, 6-minute walk test

## Discussion

HF is a common cardiac syndrome. It is estimated that there are 26 million people with HF worldwide, of which about 4 million are in China [45, 46]. HF not only affects the quality of life and life expectancy of patients, but also brings huge economic burden to the health care system [47]. Data from the urban employee medical insurance in China show that HF patients in China are mainly middle-aged and elderly, and the hospitalization rate is increasing year by year, causing a heavy burden on public health in China [48, 49]. Malnutrition is a recognized risk factor for poor prognosis in patients with HF [50, 51]. The clinical manifestations of malnutrition may vary from loss of appetite and/or weight loss, to loss of muscle mass in sarcopenia with sarcopenia, to severe cardiac cachexia [52, 53]. To prevent such adverse outcomes associated with malnutrition, current clinical practice guidelines recommend initiating nutritional support during hospitalization of medical patients at risk for malnutrition [54, 55]. In 2020, the Japanese Heart Failure Society also issued a statement on nutritional management of HF patients, pointing out that nutritional intervention for HF patients, especially ADHF patients, is of great importance [56]. A study of 241 elderly patients with acute HF showed that moderate-to-severe malnutrition is an independent risk of death in patients with acute HF, and that correcting malnutrition is significant in improving the prognosis of HF and in reducing the rate of acute HF recurrence and mortality [57]. Another study also identified underweight status and malnutrition risk as direct predictors of in-hospital mortality among male HF patients [58]. When patients are malnourished, their immunity is low and they are prone to pulmonary infection. Besides, the guidelines for heart failure clearly indicate that infection is the main cause of recurrent acute heart failure [59], and some studies have shown that recurrent acute heart failure may be related to the deficiency of certain nutrients [60]. Therefore, it is necessary to monitor and manage the key nutrients.

This study was based on the relevant clinical guidelines and original literature for middle-aged and elderly ADHF patients, and the recommendation opinions and evidence were analyzed, traced and synthesized. Different types of literature were independently evaluated using the evaluation model of the Australian Joanna Briggs Institute Evidence-Based Health Care Center (2014) [61] to ensure that the evidence included was of high quality. The protocol was developed with the participation of multiple disciplines, and was accepted by the members of the implementation team from various disciplines, and was easy to be applied in clinical practice. The protocol was refined and improved through expert consultation. The experts consulted were from tertiary hospitals in Shanghai, Suzhou, and Anhui, covering various aspects of evidence-based practice, including clinical nursing staff, nursing management staff, clinical doctors, nutritionists, etc. All experts in this study had good disciplinary representation and academic authority, as well as rich clinical experience. The participation rates of the two rounds of expert consultation were 86.7% and 100%, respectively, both > 70%. Specific modification suggestions were proposed by eleven experts and were adopted, indicating that the experts participated actively, the dialectical thinking of the evidence-based expert group was exerted, and the scientificity of the protocol was ensured. In addition, the overall authority coefficients Cr of the two rounds of experts in this study were 0.87 and 0.89, respectively. The expert authority coefficient Cr was an important analysis indicator for the reliability of the results of the Delphi expert consultation method [62]. The higher the Cr value, the higher the authority of the expert [63]. Therefore, the experts sampled in this study had authority and representativeness, and the consultation results were highly reliable.

This study aims to develop a nutrition support therapy protocol based on clinical problem-oriented approach, which has important practical significance. In the process of protocol development, we adopted standardized assessment, implementation, and evaluation procedures, and provided continuous and systematic nutrition support for patients with recurrent acute heart failure from the aspects of volume management, energy management, dietary pattern, and nutrient support. The protocol mainly includes the following measures: First, volume management is to intervene in water intake and fluid replenishment precisely from the admission of the patient, to prevent the occurrence of circulatory congestion from the source, and to reduce the risk of recurrent heart failure. Recurrent acute heart failure is often caused by systemic and pulmonary congestion, and clinically treated with diuretics, coronary vasodilators, sedatives and other drugs. Second, energy management is to estimate the energy requirement reasonably according to the patient’s metabolic status and activity level, and to adjust the energy supply timely according to the monitoring results. Third, dietary pattern is to implement individualized therapeutic diet for different comorbidities, such as Mediterranean diet, DASH diet pattern, etc. Mounting evidence underscores the potential cardiovascular advantages of plant-based diets and dietary patterns, characterized by a high consumption of plant-derived foods and minimal intake of animal products [64]. However, considering that plant-based diets are clinically associated with gastrointestinal bloating—a condition prevalent in patients with heart failure, which can impede nutritional absorption—such diets have not been incorporated into the in-hospital nutritional support protocols for patients with ADHF. Finally, nutrient support is to measure the relevant nutrients regularly according to the patient’s nutrient deficiency and tolerance, and to supplement the deficient nutrients according to the doctor’s orders: high-quality protein, amino acids, unsaturated fatty acids, minerals (potassium, sodium, iron), coenzyme Q10, vitamins, dietary fiber, probiotics, etc. The protocol is based on the guidelines of various countries and combined with the actual situation in China, to optimize and improve the protocol to make it easier to implement. For example, the guidelines recommend that registered dietitians participate in the patient’s nutrition assessment and guidance throughout the process. However, there is a shortage of clinical dietitians in reality, so this study suggests that specialized nutrition nurses complete the task, and consult dietitians when necessary.

Besides, HF patients often have comorbidities such as hypertension, hyperlipidemia or diabetes, which affect the patient’s prognosis and quality of life. Frailty and sarcopenia are two prevalent comorbidities associated with HF, with estimated prevalences in elderly HF patients of 44.5% and 34.0%, respectively [65, 66]. Both conditions are linked to increased mortality and/or hospitalization rates among HF patients [67, 68]. It is postulated that HF, frailty, and sarcopenia share numerous pathophysiological traits, including metabolic dysregulation, systemic inflammation, mitochondrial dysfunction, oxidative stress, and elevated levels of interleukin-6 [69]. These endocrine and metabolic disturbances can lead to cardiac changes and a loss of muscle mass and function, creating a vicious cycle of disability. Due to the lack of sufficient evidence supporting the use of nutritional interventions to mitigate the risks of sarcopenia and frailty in HF patients, this study did not adjust the diet specifically for HF-related sarcopenia and frailty, which is a limitation of this research. Further trials are warranted to explore the effects of isolated or combined nutritional sources on muscle mass and physical performance in heart failure patients.

## Conclusion

This study developed a nutrition support protocol for hospitalized elderly patients with ADHF, which includes four aspects: team organization and management, nutrition screening and assessment, nutrition intervention measures, and outcome evaluation. The expert consultation was conducted by using the Delphi method, which verified the rationality and scientific basis of the protocol. The research group will further conduct clinical controlled trials to verify the effectiveness and sensitivity of the protocol, and further improve the protocol content according to the actual results, to make it more applicable to clinical practice. In addition, nutrition support also requires the cooperation of the patients and their families, and the nutrition guidance for the patients and their families also needs to be paid attention to in the future.

## Data Availability

The datasets used and/or analyzed during the current study are available from the corresponding author on reasonable request.
